# De novo biosynthesis of 2-hydroxyterephthalic acid, the monomer for high-performance hydroxyl modified PBO fiber, by enzymatic Kolbe–Schmitt reaction with CO_2_ fixation

**DOI:** 10.1186/s13068-023-02413-0

**Published:** 2023-11-20

**Authors:** Yali Zhou, Shiding Zhang, Shiming Huang, Xuanhe Fan, Haijia Su, Tianwei Tan

**Affiliations:** National Energy R&D Center for Biorefnery, Beijing Key Lab of Bioprocess, College of Life Science and Technology, Beijing University of Chemical Technology, No. 15 North 3Rd Ring Rd East, Beijing, 100029 People’s Republic of China

**Keywords:** 2-Hydroxyterephthalic acid, PBO fiber, Enzymatic Kolbe–Schmitt reaction, (De)carboxylase, CO_2_ fixation, De novo biosynthetic pathway

## Abstract

**Background:**

High-performance poly(p-phenylenebenzobisoxazole) (PBO) fiber, with excellent mechanical properties (stiffness, strength, and toughness), high thermal stability combined and light weight, are widely employed in automotive and aerospace composites, body armor and sports goods. Hydroxyl modified PBO (HPBO) fiber shows better photostability and interfacial shear strength. 2-Hydroxyterephthalic acid (2-HTA), the monomer for the HPBO fiber, is usually synthesized by chemical method, which has poor space selectivity and high energy consumption. The enzymatic Kolbe–Schmitt reaction, which carboxylates phenolic substrates to generate hydroxybenzoic acids with bicarbonate/CO_2_, was applied in de novo biosynthesis of 2-HTA with CO_2_ fixation.

**Results:**

The biosynthesis of 2-HTA was achieved by the innovative application of hydroxybenzoic acid (de)carboxylases to carboxylation of 3-hydroxybenzoic acid (3-HBA) at the *para*-position of the benzene carboxyl group, known as enzymatic Kolbe–Schmitt reaction. 2,3-Dihydroxybenzoic acid decarboxylase from *Aspergillus oryzae* (2,3-DHBD_Ao) were expressed in recombinant *E. coli* and showed highest activity. The yield of 2-HTA was 108.97 ± 2.21 μg/L/mg protein in the whole-cell catalysis. In addition, two amino acid substitutions, F27G and T62A, proved to be of great help in improving 2,3-DHBD activity. The double site mutation F27G/T62A increased the production of 2-HTA in the whole-cell catalysis by 24.7-fold, reaching 2.69 ± 0.029 mg/L/mg protein. Moreover, de novo biosynthetic pathway of 2-HTA was constructed by co-expression of 2,3-DHBD_Ao and 3-hydroxybenzoate synthase Hyg5 in *S. cerevisiae* S288C with *Ura3*, *Aro7* and *Trp3* knockout. The engineered strain synthesized 45.40 ± 0.28 μg/L 2-HTA at 36 h in the CO_2_ environment.

**Conclusions:**

De novo synthesis of 2-HTA has been achieved, using glucose as a raw material to generate shikimic acid, chorismic acid, and 3-HBA, and finally 2-HTA. We demonstrate the strong potential of hydroxybenzoate (de)carboxylase to produce terephthalic acid and its derivatives with CO_2_ fixation.

**Supplementary Information:**

The online version contains supplementary material available at 10.1186/s13068-023-02413-0.

## Background

CO_2_ is the primary greenhouse gas and results in global warming, which comes from the burning of fossil fuels and forests. Using CO_2_ as a C1 carbon source to synthesize value-added chemicals and materials is an attractive strategy to reduce atmospheric CO_2_. While CO_2_ is chemical stability and high energy is required to activate this substrate.

The Kolbe–Schmitt reaction is a famous CO_2_ fixation process, which carries out carboxylation of phenol at the *ortho-* and *para-*position at 125 °C, 100 atm [1]. The enzymatic Kolbe–Schmitt reaction provides an effective approach to carboxylate phenolic substrates under a mild condition (bicarbonate/CO_2_, 30 ℃) and employs nonoxidative decarboxylase which is originally discovered to catalyze the decarboxylation of benzoic acid derivatives. These hydroxybenzoic acid (de)carboxylases have exquisite regioselectivity in *ortho*-position of the phenolic -OH group and have a remarkably broad substrate spectrum that phenol derivatives containing alkyl, alkoxy, halo and amino groups are tolerated [2, 3]. However, the reverse carboxylation reaction is less favorable than decarboxylation. To push the reaction equilibrium towards the carboxylation, quaternary ammonium salts are added and the yields of the carboxylation products are increased from 40% to 97% [4]. In addition, pressurized CO_2_ gas (∼ 30–40 bar) is used instead of bicarbonate and the carboxylation process is greatly promoted, as the carboxylation conversion of resorcinol is up to 68% [5].

Nature CO_2_ fixation pathways are well-researched and the CO_2_ fixation enzymes are applied to construct the new biosynthetic pathways [6–8]. Propionyl-CoA carboxylase (PCC) is expressed to construct the succinate biosynthetic pathway in recombinant *Escherichia coli* (*E. coli*), which produces succinate from acetyl-CoA with the fixation of two CO_2_ molecules [9]. Methanol-assimilation pathway is designed into a CO_2_ Calvin–Benson–Bassham cycle that converts the yeast *Pichia pastoris* into an autotroph of capable growing with CO_2_ as a sole carbon source [10]. Hydroxybenzoic acid (de)carboxylases have the potential to construct the biosynthetic pathway of aromatic acids, which have been used as raw materials for medicines, flavors and plastics.

This study focuses on the production of 2-hydroxyterephthalic acid (2-HTA), the monomer for high-performance poly(hydroxy-p-phenylenebenzobisoxazole) (HPBO) fiber. It is known that poly(p-phenylene benzobisoxazole) (PBO) fiber has excellent thermal stability (up to 650 °C), outstanding mechanical properties (tensile strength of 5.8 GPa, modulus of 270 GPa), low density (1.56g/cm^3^) [11], and has been used as an ideal reinforcement for aerospace, defense, military and other fields. The main shortcomings of PBO fiber are poor interfacial adhesion and compatibility, photodegradation under UV exposure. HPBO fiber is prepared by copolymerization of 4,6-diaminoresorcinol dihydrochloride (DARH) and 2-HTA. It maintains thermal stability (up to 600 ℃), mechanical properties (tensile strength of 4.32 GPa, modulus of 169.7 GPa) while improving the compression resistance, composite bonding properties and photostability due to the presence of hydroxyl groups in polymer chains [12, 13]. Besides, 2-HTA is a raw material of hypercrosslinked polymers (HCPs) [14] and metal–organic frameworks (MOFs) [15, 16].

Traditionally, 2-HTA is synthesized by chemical synthesis, including 2-bromoterephthalic acid (BTA) catalytic hydrolysis, and copper powder catalyzes BTA to generate 2-HTA at 100 °C in an alkaline environment; Koble–Schmitt method, 3-hydroxybenzoic acid (3-HBA) is carboxylated to 2-HTA at 230–240 ℃ in the presence of K_2_CO_3_/CO_2_ (atmospheric) and potassium formate solvent [12]. Biosynthesis combining chemical synthesis has been reported to produce 2-HTA: a *Pseudomonas* mutant was screened to oxidize Terephthalic acid (TPA) to a diol intermediate, 1,2-dihydroxycycclohexa-3,5-diene-1,4-dicarboxylic acid, and then the diol intermediate was dehydrated by sulfuric acid to form 2-HTA [17]. The chemical process exhibits non-specific regioselectivity with carboxylation at both *ortho-* and *para-*positions and requires high temperature. However, biosynthesis of 2-HTA has not been reported.

In this study, we use the enzymatic Kolbe–Schmitt reaction to convert 3-HBA to 2-HTA. It is the first report to produce 2-HTA with these (De)carboxylases. Due to the low carboxylation activity towards 3-HBA, mutants obtained by site-directed mutagenesis are used to enhance catalytic activity. The chorismate lyase (XanB2) and 3-hydroxybenzoate synthase (Hyg5) have been reported to use chorismate as a precursor to synthesize 3-HBA [18, 19]. Both Hyg5 and (De)carboxylases for the enzymic Kolbe–Schmitt reaction are applied in the construction of biosynthetic pathways to produce 2-HTA with 3-HBA as the intermediate in recombinant *Saccharomyces cerevisiae*. By culturing the recombinant strain in the CO_2_ gas, the de novo synthesis of 2-HTA based on the shikimic acid pathway is achieved using glucose as a raw material.

## Results and discussion

### Whole-cell reaction with new substrate 3-HBA

Hydroxybenzoic acid (de)carboxylases is a reversible nonoxidative decarboxylases which has been used in Kolbe–Schmitt reaction with CO_2_ fixation. The family members including 2,6-dihydroxybenzoate decarboxylase (2,6-DHBD) from *Rhizobium sp.* (2,6-DHBD_Rs), 2,3-dihydroxybenzoic acid decarboxylase (2,3-DHBD) from *Aspergillus oryzae* (2,3-DHBD_Ao) [20], 2,3-DHBD from *Fusarium oxysporum*, (2,3-DHBD_Fo) [21, 22], salicylic acid decarboxylase (SAD) from *Trichosporon moniliiforme* (SAD_Tm) [23, 24] show exclusive regioselectivity for carboxylation in the *ortho*-position of the aromatic phenolic group. 2,6-DHBD_Rs has a narrow substrate spectrum and has no activity on monohydric phenols, such as phenol. 2,3-DHBD_Ao and SAD_Tm have broad substrate spectrum [25], only require the presence of phenolic hydroxyl group, and are tolerant to alkyl, alkoxy, halogen, and amino groups [2, 3]. This study is the first attempt to use this kind of enzymes to add a carboxyl group to the *para-*position of aromatic carboxylic acids to form terephthalic acid derivatives. We use 3-HBA as a substrate to generate 2-HTA.

Lyophilized *E. coli* BL21(DE3) whole cells expressing hydroxybenzoic acid (de)carboxylases catalyzed the natural substrate resorcinol to generate 2,4-dihydrobenzoic acid and 2,6-dihydrobenzoic acid in vitro, in which 2, 6-dihydrobenzoic acid was more than 2, 4-dihydrobenzoic acid. 2,3-DHBD showed better activity to resorcinol than SAD_Tm (Additional file 1: Table S1). Thus, 2,3-DHBD_Ao and 2,3-DHBD_Fo were selected to catalyze 3-HBA.

**Fig. 1:**
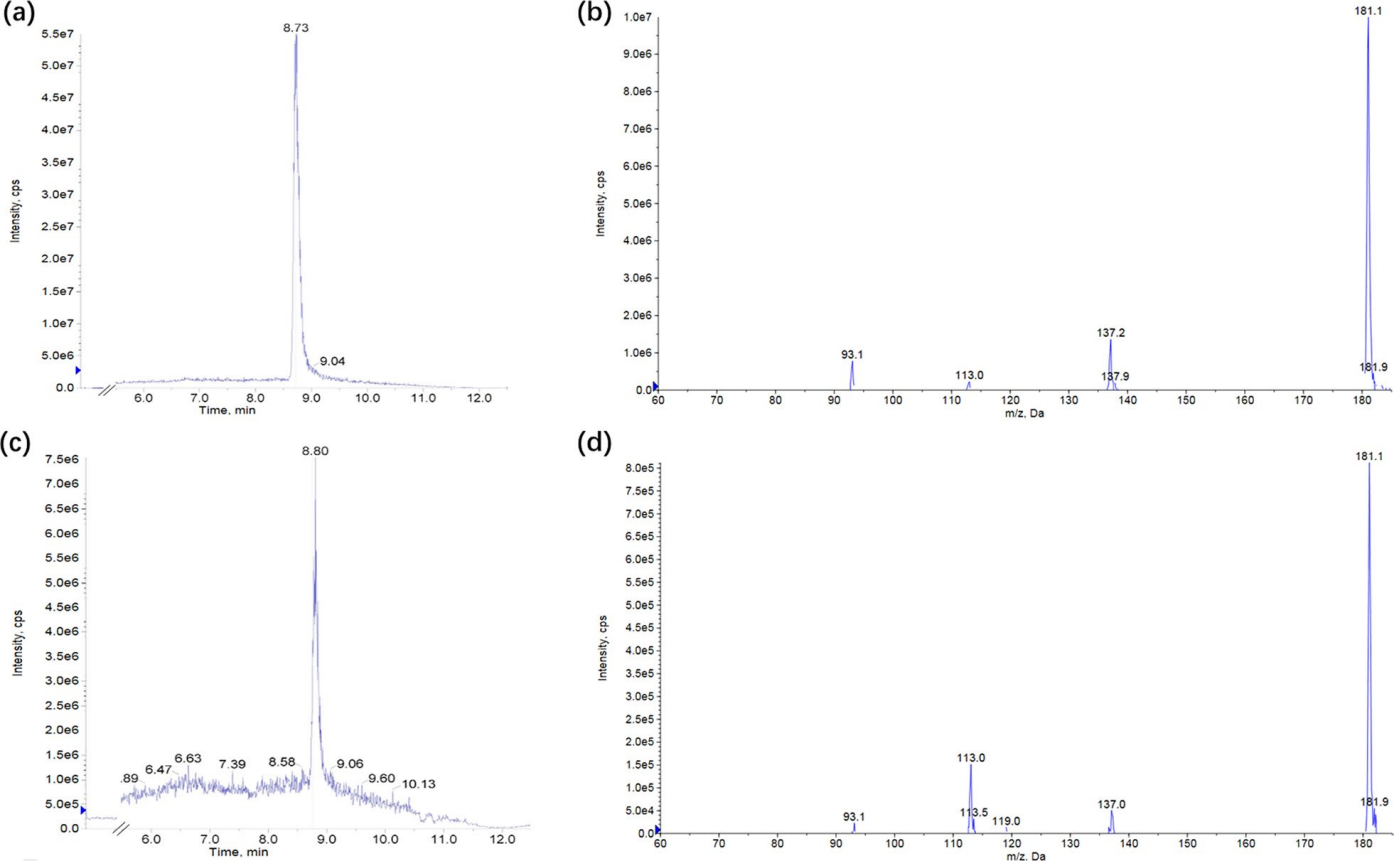
HPLC–MS/MS analysis of 2-HTA and whole-cell reaction mixture of 2,3-DHBD_Ao. **a** Extracted ion chromatography of 2-HTA standard. **b** Secondary mass spectrum of 2-HTA standard. **c** Extracted ion chromatography of whole-cell reaction mixture of 2,3-DHBD_Ao. **d** Secondary mass spectrum of whole-cell reaction mixture of 2,3-DHBD_Ao

Reaction mixtures of 2,3-DHBD_Ao were analyzed by HPLC–MS/MS. A specific peak with the same retention time as the 2-HTA standard was detected in the reaction mixture. Dissociation of the 181.1 Da [M–H]^−^ ion resulted in major ion fragments 137.2 and 93.1 Da with one and two CO_2_ losses, respectively, the same with 2-HTA standard (Fig. 1). Furthermore, the quantitation of 2-HTA was carried out with an ion of m/z 137.2 in the multiple reaction monitoring (MRM) model.

**Fig. 2:**
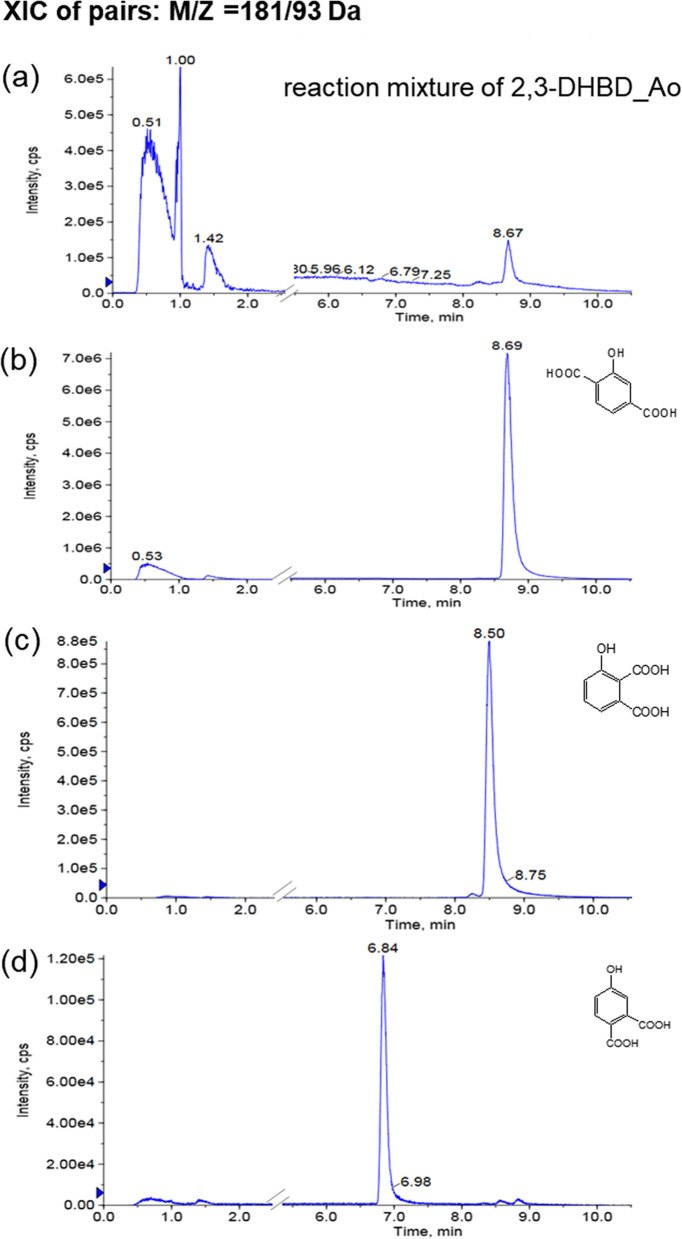
HPLC–MS/MS analysis: **a** whole-cell reaction mixture of 2,3-DHBD_Ao, **b** 2-HTA standard, **c** 3-hydroxyphthalic acid standard, **d** 4-hydroxyphthalic acid standard

To identify whether 3-HBA generates multiple products, some potential products, such as 2-HTA, 3-hydroxyphthalic acid standard and 4-hydroxyphthalic acid, were detected by HPLC–MS/MS. The XIC of pairs of 181/93 Da showed that whole-cell reaction mixture, 2-HTA, 3-hydroxyphthalic acid, and 4-hydroxyphthalic acid peaked at 8.67, 8.69, 8.50, and 6.84 min, respectively. The retention time of 3-HBA reaction mixture was consistent with that of 2-HTA standard (Fig. 2). It was proved that 3-HBA generates 2-HTA rather than other isomers under the catalysis of 2,3-DHBD.

**Fig. 3:**
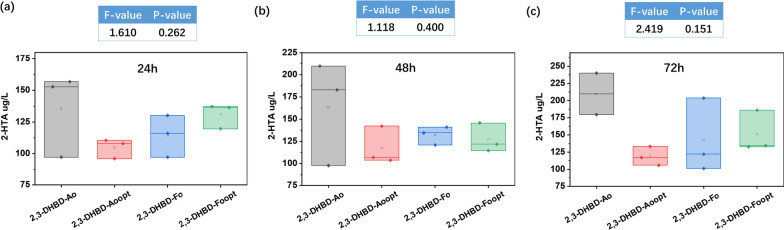
2-HTA production by whole-cell reaction of *E. coli* BL21(DE3) harboring different 2,3-DHBD. **a** Reaction conditions: 30 mg/mL lyophilized whole cells, 10 mM substrate, 3 M KHCO_3_, 30 ℃, 180 rpm. **b** Enzyme: 2,3-DHBD_Ao_opt_: 2,3-DHBD_Ao with codon-optimized; 2,3-DHBD_Fo_opt_: 2,3-DHBD_Fo with codon-optimized; sequences of native and codon-optimized genes were shown in Additional file 1: Table S3. **c** One-way analysis of variance (one-way ANOVA) was used to analyze the data

As shown in Fig. 3, both 2,3-DHBD_Ao and 2,3-DHBD_Fo had carboxylation activity for 3-HBA. There was no significant difference on carboxylation activity whether codon optimization was performed or not. Codon optimization had effect on the expression of enzyme. 2,3-DHBD_Ao with codon optimization expressed the least protein (Additional file 1: Table S2). Thus, 2,3-DHBD_Ao without codon-optimized was chosen to produce 2-HTA, the yield of 2-HTA is 108.97 ± 2.21 μg/L/mg protein.

### Site-directed mutagenesis of 2,3-DHBD_Ao

**Fig. 4:**
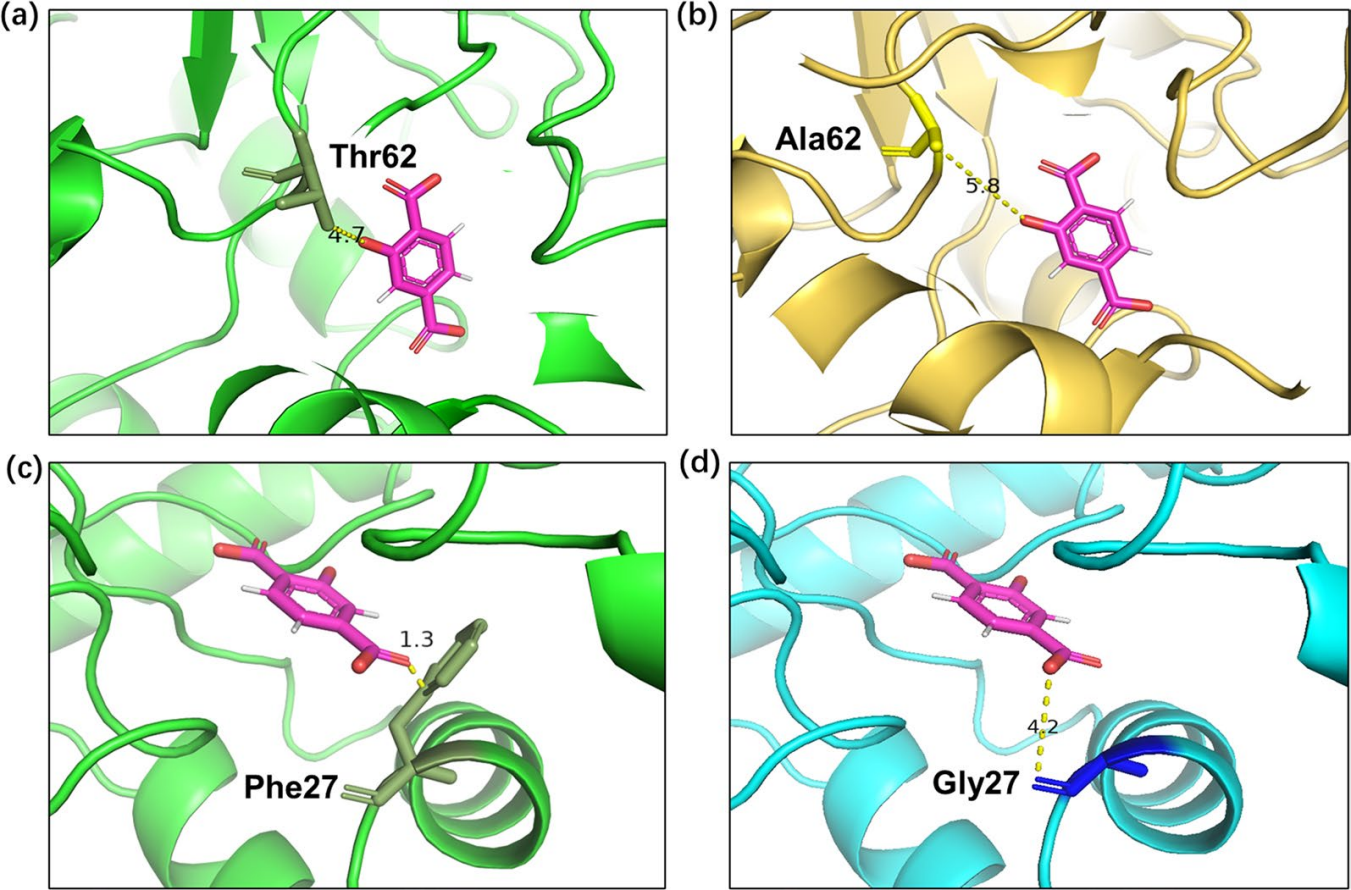
Comparison of distances between residues 62/27 and 2-HTA. **a** Wild-type 2,3-DHBD_Ao (PDB ID:7WKM) with Thr62 (green stick); **b** 2,3-DHBD_Ao mutant with Ala62(yellow stick) ^a^; **c** wild-type 2,3-DHBD_Ao with Phe27 (green stick); **d** the 2,3-DHBD_Ao mutant with Gly27 (blue stick) ^a^. ^a^ Mutants were generated by Discovery Studio™ based on 7WKM

**Fig. 5:**
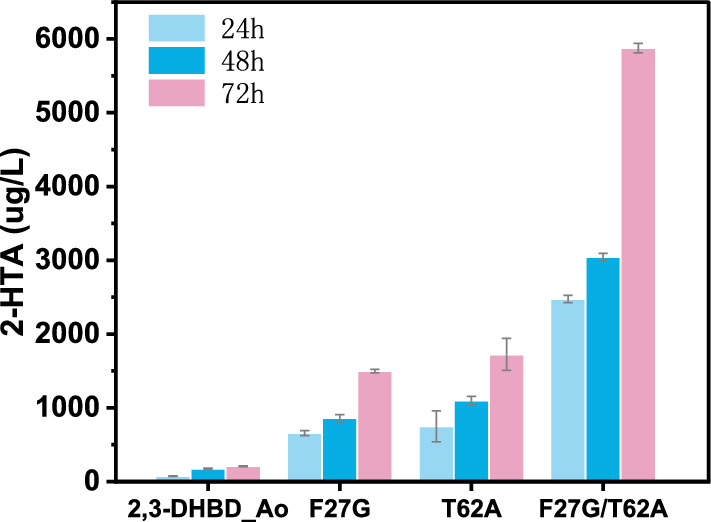
2-HTA production by 2,3-DHBD_Ao mutants with single and double amino acid substitutions. ^a^Reaction conditions: 30 mg/mL lyophilized whole cells, 10 mM substrate, 3 M KHCO_3_, 30 ℃, 180 rpm

Hydroxybenzoic acid (de)carboxylases from microbial sources have a very similar structure. It exists as a homotetramer, each subunit is a (β/α)8 barrel, the active site has a divalent metal ion, and the reaction mechanism is similar [26–30]. The literature revealed the steric hindrance of the substrate access channel in SAD_Tm enzyme and enlarged the substrate-binging pocket to accelerate the substrate to the active metal ion. Thus, the activity increased by 26.4-fold [31]. Amino acid residue T62 of 2,3-DHBD_Ao was not conserved. Mutation at this site proved to be useful [31, 32]. In this study, mutation of threonine-62 to alanine was implemented to reduce steric hindrance of substrate-binging pocket. On the other side, phenylalanine-27 was found to be very close to the carboxyl group of 2-HTA, so phenylalanine-27 was mutated to glycine to reduce steric hindrance (Fig. 4). These two single mutants of 2,3-DHBD_Ao were constructed by site-directed mutagenesis. F27G and T62A were of assistance in carboxylation activity improvement, showing 6.3-fold and 8.2-fold higher than the wild type. Double mutant F27G/T62A produced the highest concentration of 2-HTA in the whole-cell reaction with 5875 ± 63.64 μg/L at 72 h (Fig. 5). The yield of 2-HTA was 2.69 ± 0.029 mg/L/mg protein and 24.7 times higher than the wild type. Pure enzymatic catalysis of the mutants showed increased enzyme activity (Additional file 1: Fig. S2).

### Strains resistant to KHCO_3_

In general, saturated KHCO_3_ was used in the enzyme carboxylation reaction in vitro. To verify whether the strain could grow normally in the KHCO_3_ solution, KHCO_3_ tolerance experiments were carried out on different strains.

*E. coli* strains BL-0 and BL-A were used in the tolerance experiment. OD600 of BL-A first increased and then decreased in 0.5 M KHCO_3_ LB medium, at last remained at about 0.6. The OD_600_ of BL-0 decreased continuously in 1 M and 0.5 M KHCO_3_ LB medium. These proved that 1 M and 0.5 M KHCO_3_ inhibited the growth of *E. coli* (see Table 1)*.*

**Table 1:** OD_600_ of *E. coli* (BL-0, BL-A) in LB medium with different concentrations of KHCO_3_

KHCO_3_	1 M			0.5 M		
	0 h	17 h	35 h	0 h	17 h	35 h
BL-0	0.767 ± 0.027	0.308 ± 0.025	0.249 ± 0.045	0.672 ± 0.02	0.400 ± 0.055	0.300 ± 0.035
BL-A	0.578 ± 0.037	0.231 ± 0.018	0.187 ± 0.023	0.563 ± 0.042	0.719 ± 0.033	0.577 ± 0.029

**Fig. 6:**
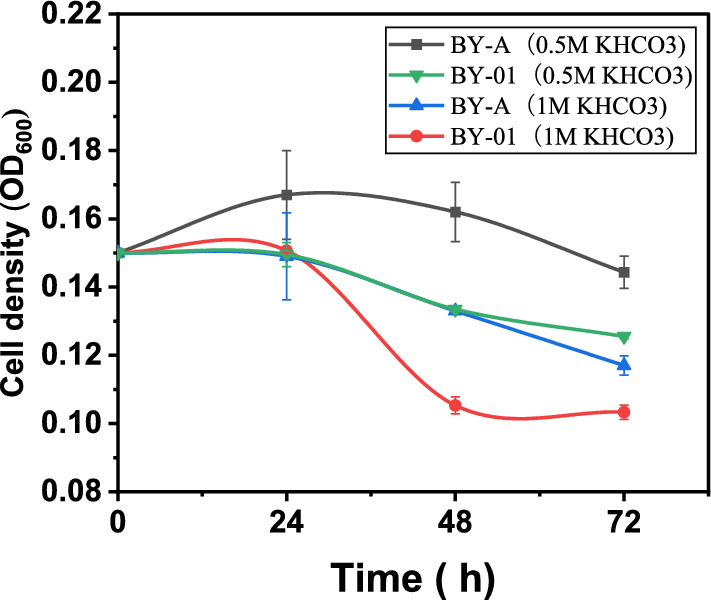
OD_600_ of *S. cerevisiae* BY4741 in 1 M and 0.5 M KCHO_3_ medium

*Saccharomyces cerevisiae* strains BY-4741 (BY-01 and BY-A) were cultured in SC medium with 1 M and 0.5 M KHCO_3_. BY-01 and BY-A have an initial OD600 of 0.15. In 1 M and 0.5 M KHCO_3_, the growth of strains was inhibited, and OD_600_ was between 0.10 and 0.17 (Fig. 6). The pH value of 1 M KHCO_3_ medium was 7.38 at 72 h, the pH value of 0.5 M KHCO_3_ medium was 7.32. Yeast can grow normally at pH 7, indicating that the growth inhibition by KHCO_3_ is not caused by pH.

### Carboxylation at different concentrations of KHCO_3_

Enzymatic carboxylation was performed at different concentrations of KHCO_3_. The effect of reducing the concentration of KHCO_3_ on the enzymatic carboxylation was investigated to find the appropriate concentration of KHCO_3_ that can not only make the strain grow but also allows the carboxylation to proceed normally.

BL-A was used to catalyze the carboxylation of resorcinol. Under different concentrations of KHCO_3_ (3, 0.5, 0.1 M), the selectivity of 2,6-dihydroxybenzoic acid decreased as the concentration of KHCO_3_ declined. The selectivity was only 13.7% when the KHCO_3_ concentration dropped to 0.5 M. No 2,6-dihydroxybenzoic acid was produced, when the concentration of KHCO_3_ dropped to 0.1 M (Table 2).

**Table 2:** Effect of KHCO_3_ concentration on the production of 2,6-dihydroxybenzoic acid from resorcinol

Entry	KHCO_3_	2,6-Dihydroxybenzoic acid Selectivity (%)^a^
1	3 M	55.68
2	0.5 M	13.70
3	0.1 M	0

^a^Reaction conditions: 20 mg/mL lyophilized whole cells expressed 2,3-DHBD_Ao, 10 mM resorcinol, 30 ℃, 180 rpm, 48 h

**Fig. 7:**
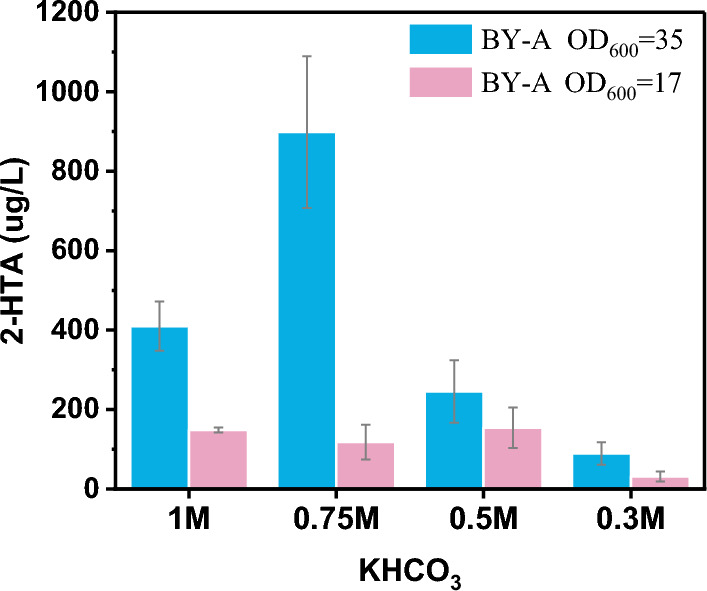
Effect of KHCO_3_ concentration on the production of 2-HTA by *S. cerevisiae* BY-A. ^a^ Reaction conditions: 10 mM 3-HBA, 30 ℃, 180 rpm, 72 h

*Saccharomyces cerevisiae* BY4741 strain BY-A with an initial OD_600_ of 35 (group 1) and 17 (group 2) decreased sharply in the production of 2-HTA in the 0.3 M KHCO_3_. Compared with 0.5 M KHCO_3_, 2-HTA of group 1 decreased by 77.46%, and group 2 decreased by 79.55% (Fig. 7).

The growth of *E. coli* and yeast strains was inhibited in 0.5 M KHCO_3_ solution. However, when the concentration of KHCO_3_ was lower than 0.5 M, the efficiency of the carboxylation reaction dropped sharply. It was difficult to find a balance.

### De novo synthesis of 2-HTA in *S. cerevisiae* using CO_2_ as carboxylation reagent

In the enzymatic carboxylation reaction, not only can KHCO_3_ be used as the carboxylation reagent, but there are also reports that pressurized CO_2_ (30–40 bar) is used to replace KHCO_3_ for enzyme-catalyzed carboxylation. The pressure of CO_2_ has a great influence on carboxylation efficiency. In the literature, resorcinol was catalyzed by 2,3-DHBD_Ao (10 mM substrate concentration, 100 mM pH 9.0 TRIS–HCl buffer), and the conversion reached 68%. As the CO_2_ pressure decreased, the conversion rate also decreased. Especially when the pressure was less than 20 bar, the conversion rate decreased sharply. At 10 bar, the conversion rate was very low, less than 20% [5].

In addition, pH is also an important parameter affecting the reaction. The stability of 2,3-DHBD_Ao and 2,6-DHBD_Rs is affected by pH. These two enzymes are active between pH 4–9, and the thermal stability is the highest between pH 6–7. In a low pH environment, the denaturation temperature decreases. Below pH 4.6, the enzyme is unstable at room temperature.

The previous experiments have proved that the low concentration of KHCO_3_ will reduce the carboxylation efficiency, while a high concentration of KHCO_3_ will inhibit the growth of the strain. Therefore, CO_2_ is intended to replace KHCO_3_ as the carboxylation reagent in this experiment.

**Fig. 8:**
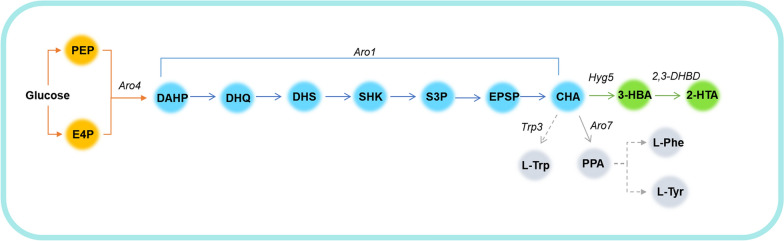
Biosynthesis pathway of 2-HTA. PEP, phosphoenolpyruvate; E4P, erythrose 4-phosphate; DAHP, 3-deoxy-D-arabinoheptulosonate 7-phosphate; DHQ, 3-dehydroquinate; DHS, 3-dehydroshikimate; SHK, shikimate; S3P, shikimate-3-phosphate; EPSP, 5-enolpyruvyl-shikimate 3-phosphate; CHA, chorismate; PPA, prephenate; L-Phe, L-phenylalanine; L-Trp, L-tryptophan; L-Tyr, L-tyrosine; *Aro1*, pentafunctional aromatic protein; *Aro4,* DAHP synthase; *Hyg5*, 3-hydroxybenzoate synthase; *2,3-DHBD*, 2,3-dihydroxybenzoate decarboxylase; *Aro7*, chorismate mutase; *Trp3*, indole-3-glycerol-phosphate synthase

In addition, *S. cerevisiae* converts one glucose molecule to two molecules of CO_2_ in the fermentation process that is beneficial to form the CO_2_ environment required for carboxylation. *S. cerevisiae* was chosen for the experiment. Hyg5 and (de)carboxylase were introduced exogenously, combined with the strain's shikimate pathway to construct a 2-HTA production pathway (Fig. 8).

The *S. cerevisiae* strains CEN.PK113-5D 5D-H expressing Hyg5 and 5D-HA expressing Hyg5 and 2,3-DHBD_Ao were used for fermentation. At the same time, the growth and metabolism of yeast strains in CO_2_ environment (CO_2_ group) and ordinary air environment (O_2_ group) were compared.

**Fig. 9:**
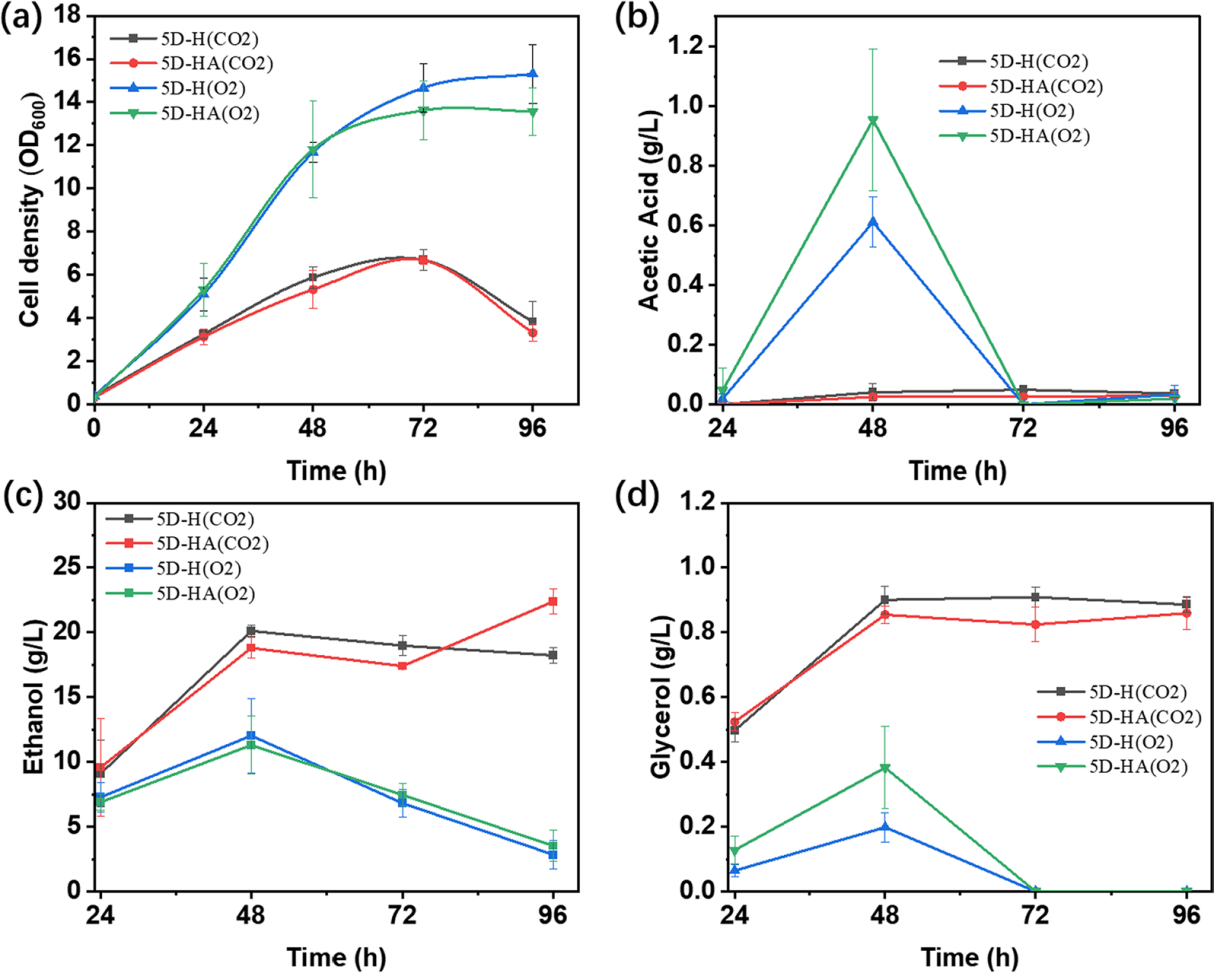
Fermentation comparison of *S. cerevisiae* CEN.PK113-5D under CO_2_ and O_2_ environment. **a** Strain growth curve. **b** Accumulation of acetic acid, **c** accumulation of ethanol, **d** accumulation of glycerol

**Fig. 10:**
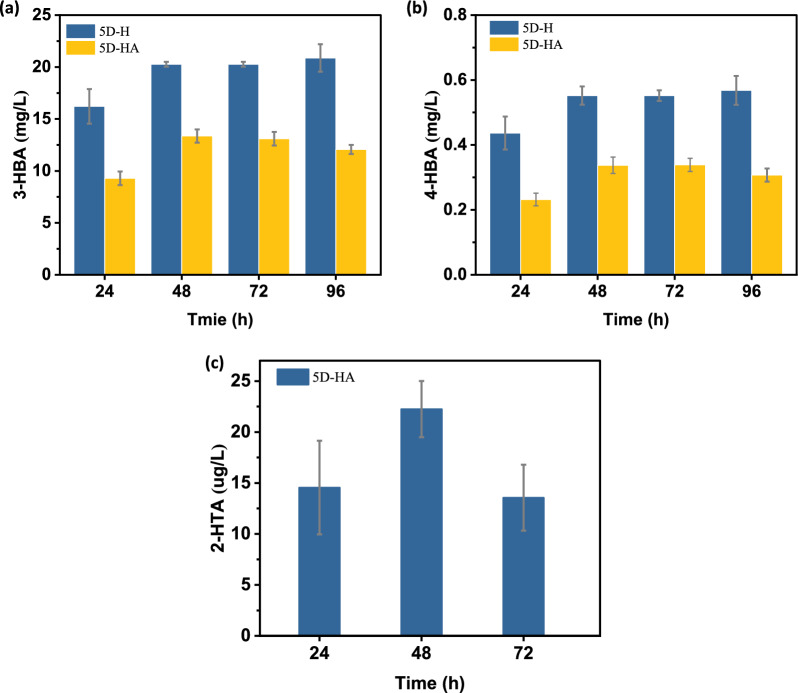
Fermentation of *S. cerevisiae* CEN.PK113-5D in SC medium under CO_2_ environment. **a** Concentration of 3-HBA. **b** Concentration of 4-HBA. **c** Concentration of 2-HTA

In the O_2_ group, OD_600_ reached 14. Ethanol reached the highest value of 12 g/L at 48 h, and then gradually decreased to 2.5 g/L at 96 h. Glycerol was ultimately metabolized without accumulation. In the CO_2_ group, OD_600_ was only 6. Ethanol accumulated to a concentration 20 g/L, and glycerol to 0.9 g/L. In these two groups, only small amounts of acetic acid were detected (Fig. 9). The concentration of 2-HTA in the CO_2_ group was 22.25 ± 2.75 μg/L at 48 h (Fig. 10).

The strain 5D-H produced 20.27 ± 0.23 mg/L of 3-HBA at 48 h, and the strain 5D-HA co-expressing carboxylase decreased the 3-HBA concentration to only 13.10 ± 0.65 mg/L at 48 h. In this experiment, it was found for the first time that the expression of the Hyg5 can produce 3-HBA and 4-HBA. 4-HBA concentration is 0.55 ± 0.04 mg/L for 5D-H and 0.34 ± 0.02 mg/L for 5D-HA at 48 h (Fig. 10).

**Fig. 11:**
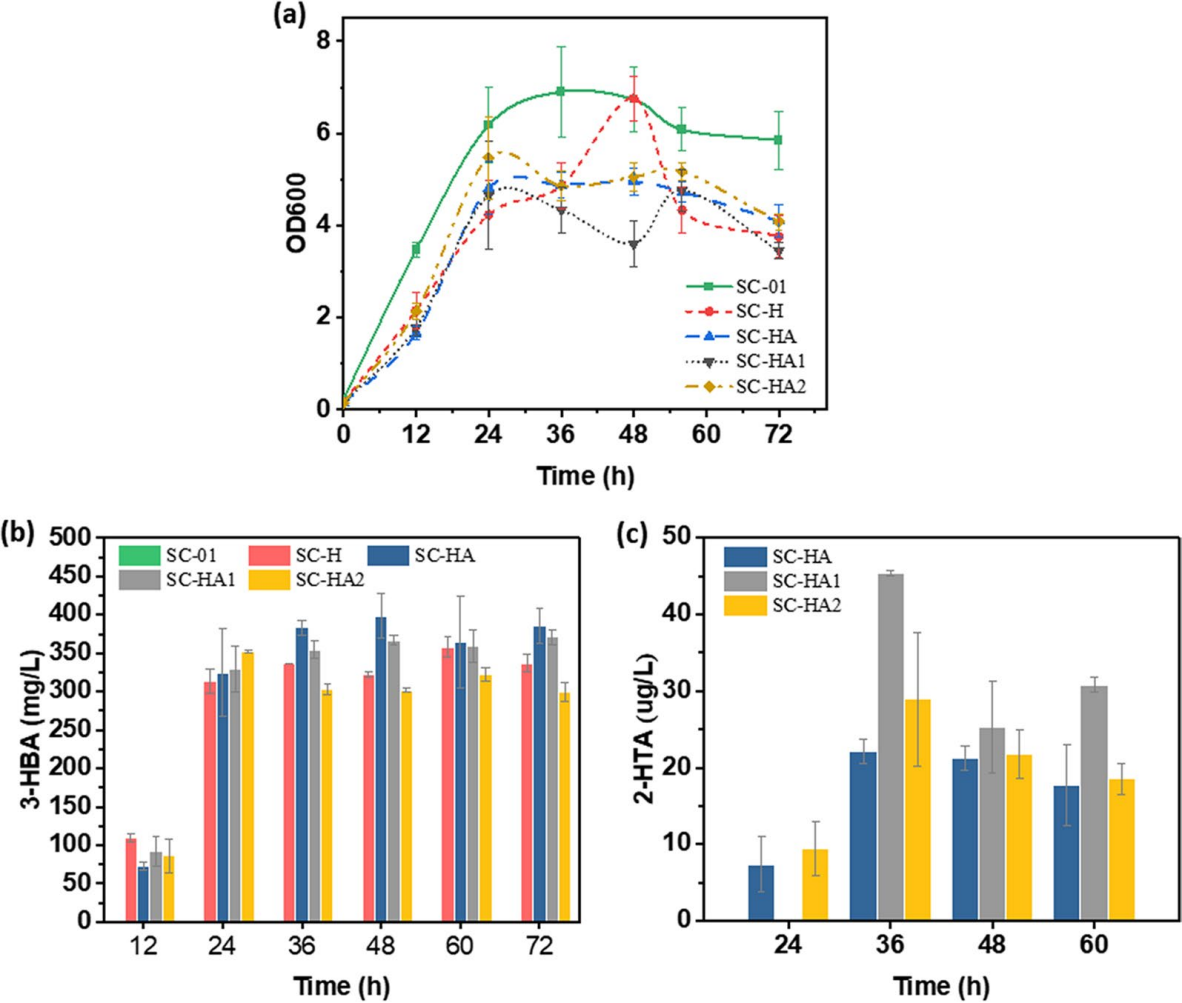
Production of *S. cerevisiae* S288C in SC medium under CO_2_ environment. **a** Growth curve. **b** Concentration of 3-HBA. **c** Concentration of 2-HTA

The production of 2-HTA in *S. cerevisiae* requires the diversion of carbon flux towards 3-HBA synthesis through elimination of competing metabolic pathways and enhancement of 3-HBA synthesis. A strategy has been reported that combines feedback-resistant *Aro4*^*K229L*^ and deletion of the *Aro7* and *Trp3* genes to direct flux to chorismite [33]. *S. cerevisiae* strain S288C with *Ura3*, *Aro7* and *Trp3* knocked out (S288C-AT) was used as chassis strain. The strains SC-01, SC-H, SC-HA, SC-HA1 and SC-HA2 (containing pSP-GM1, Hyg5, Hyg5 and 2,3-DHBD_Ao, hyg5 and 2,3-DHBD_Ao ^T62A^, Hyg5 and 2,3-DHBD_Ao^F27G/T62A^, respectively) were cultured in 30 mL SC medium (0.05 M KHCO_3_). The initial OD_600_ was about 0.2, and the final OD_600_ was maintained at 5. The 3-HBA yields of SC-HA, SC-HA1 and SC-HA2 were 398.59 ± 28.43 mg/L, 366.94 ± 6.46 mg/L, and 352.69 ± 1.67 mg/L, respectively. The SC-HA produced 22.10 ± 1.56 μg/L of 2-HTA, which was close to that of 5D-HA, and the production of 2-HTA in SC-HA1 increased to 45.40 ± 0.28 μg/L (Fig. 11). In addition, the yield of 2-HTA in SC-HA2 expressing mutant 2,3-DHBD_Ao^F27G/T62A^ was basically the same as that of SC-HA, without any increase. The 3-HBA production of SC-HA2 was lower than that of SC-HA and SC-HA1, so it was speculated that the same reason led to the failure to increase the production of 2-HTA in SC-HA2.

## Conclusion

Hydroxybenzoic acid (de)carboxylases can catalyze the reversible decarboxylation reaction that converts phenols to hydroxybenzoic acids. In this study, the substrate spectrum of these enzymes was broadened, and the enzyme 2,3-DHBD_Ao was successfully used to generate the 2-HTA, the monomer for high-performance PHBO fiber. The enzyme activity was improved by site-directed mutagenesis. In vitro, whole-cell catalysts with the single point mutation F27G and T62A increased the production of 2-HTA by 6.3-fold and 8.2-fold. The double site mutation F27G/T62A increased the production of 2-HTA by 24.7-fold up to 2.69 ± 0.029 mg/L/mg protein.

Combined with the shikimate pathway, a de novo biosynthesis pathway of 2-HTA was designed, from glucose to shikimate, chorismate, 3-HBA, and finally 2-HTA. 3-hydroxybenzoate synthase Hyg5 and hydroxybenzoic acid (de)carboxylases 2,3-DHBD_Ao^T62A^ were co-expressed in *S. cerevisiae* S288C which was knocked out of *Ura3*, *Aro7* and *Trp3*. The engineered strain synthesized 2-HTA de novo in CO_2_ with a titer of 45.40 ± 0.28 μg/L.

This experiment tried to use bicarbonate as a carboxylation reagent and found that living cells can only tolerate low concentrations of bicarbonate. The sensitivity is not caused by bicarbonate-induced changes in solution pH, but may be related to bicarbonate regulation in biological systems.

This pathway can be further developed on the productivity of TPA and its derivatives and the efficiency of carboxylases. It is also needed further research on intracellular utilization of carbon source.

## Materials and methods

### Strains and media

The strains and plasmids used in this study were listed in Additional file 1: Table S4. *E. coli* trans10 was used for propagating and storing the plasmids. *E. coli* BL21(DE3) was used for protein expression. *S. cerevisiae* strains were used for 2-HTA biosynthesis.

*E. coli* strains were cultured in Luria–Bertani (LB) broth (10 g/L tryptone, 5 g/L yeast extract, and 10 g/L NaCl). The yeast strains were cultivated in synthetic complete (SC) medium without the auxotrophic compound complemented by the plasmids, which consisted of 6.7 g/L yeast nitrogen base (YNB), 5 g/L (NH_4_)_2_SO_4_, 0.77 g/L complete supplement mixture (CSM) without uracil or histidine, 20 g/L glucose.

### Construction of plasmids

Molecular cloning was performed according to standard protocols [34]. The oligonucleotide primers for plasmid construction are listed in Additional file 1: Table S5. The genes *2,3-DHBD_Ao* (NCBI reference sequence: XM_001817461.3) encoding 2,3-dihydroxybenzoic acid decarboxylase from *Aspergillus oryzae* (2,3-DHBD_Ao), *2,3-DHBD_Fo* (GenBank: EXM04459.1) encoding 2,3-DHBD from *Fusarium oxysporum* (2,3-DHBD_Fo), *SAD_Tm* (GenBank: DM040453.1) encoding salicylic acid decarboxylase from *Trichosporon moniliiforme* (SAD_Tm) and *Hyg5* (GenBank: AF007101.1) encoding 3-hydroxybenzoate synthase from *Streptomyces hygroscopicus*, were synthesized by BGI Beijing (China), and cloned into appropriate vectors.

To construct site-directed mutation of *2,3-DHBD_Ao*, mutants were amplified from pET28a( +)-2,3-DHBD_Ao using the corresponding up and down primers, and assembled by Gibson assembly [35].

### Protein expression and carboxylation

2,3-DHBD was expressed in *E. coli* BL21(DE3). *E. coli* BL21(DE3) strain was grown in LB medium at 37 ℃ and induced with 0.2 mM IPTG at OD_600_ 1.0.

In the whole-cell carboxylation reactions, 30 mg lyophilized *E. coli* BL21(DE3) whole cells were suspended in 1 mL deionized water and rehydrated for 30 min, followed by adding substrate, 3 M KHCO_3._ The mixture was incubated at 30 ℃ for 72 h in sealed glass vials.

The reaction mixture was stopped by adding 1 mL 6 M HCl. The resulting mixture was centrifuged at 12,000 rpm for 5 min. After dilution and filtration, the sample was analyzed by HPLC and HPLC–MS.

### Analytics

HPLC analysis was performed on Dionex/Thermo Ultimate 3000 system with a UV detector at 254 nm and a Hypersil GOLD C18 (5 μm, 150 × 4.6 mm, column temperature 25 °C). The method was run over 18 min with H_2_O/trifluoroacetic acid (0.1%) as the mobile phase (flow rate 0.8 mL/min) and an acetonitrile/trifluoroacetic acid (0.1%) gradient (0–1 min 5%, 1–8 min 5–70%, 8–11 min 70%, 11–13 min 70–100%, 13–15 min 100%). Dionex/Thermo Ultimate 3000 system with an oscillometric detector was used to detect the concentration of glucose, ethanol, glycerol, and acetic acid concentration in the medium. Bio-Rad 87H column was used with 5 mM sulfuric acid as the mobile phase, a column temperature of 65 °C, and a flow rate of 0.6 mL/min.

HPLC–MS/MS analysis was performed on the Shimadzu LC 20A system with SCIEX QTRAP 5500 systems as mass-detector and Agilent Pursuit PFP (2.7 µm, 2.1 × 100 mm, column temperature 40°C). The method was run over 15min with H_2_O/formic acid (0.1%) as the mobile phase (flow rate 0.2 mL/min) and a methanol gradient (0–1 min 10%, 1–4 min 10–70%, 4–6 min 70%, 6–9 min 70–100%, 9–13 min 100%). MS was operated in the negative-ion mode with electrospray ionization source (ESI).

### KHCO_3_ tolerance experiment

Prepare LB culture medium with 0.1 M pH 6 potassium phosphate buffer instead of water. The strain BL-A expressing 2,3-DHBD_Ao and BL-0 strain expressing pET-28a( +) empty plasmid was inoculated into the LB culture medium containing 5 mM substrate 3-HBA. 1‰ IPTG was used to induce protein expression, and samples were taken at 0, 17, 35 h to measure OD_600_.

30 mL SC medium (30 g/L glucose) containing 1 M and 0.5 M KHCO_3_ was added into 100 mL anaerobic bottles and infused CO_2_ for 5 min. *S. cerevisiae* strains were inoculated and cultured at 200 rpm/min for 72 h.

### Fermentation and 2-HTA production

For 2-HTA production, strains were cultured in the 100 mL anaerobic bottle at 30 ℃ and 200 rpm/min with 30 mL CO_2_-saturated SC medium lacking the auxotrophic compound complemented by the plasmids. The metabolites were detected by HPLC and HPLC–MS/MS.

### Supplementary Information

**Additional file 1: Table S1.** Enzymic carboxylation with natural substrates. **Table S2.** Quantitative analysis of protein expression in 2,3-DHBD—Ao, Ao_opt_, Fo, Fo_opt_, Ao ^F27/T62^. **Table S3.** Codon-optimized DNA sequences. **Table S4.** Strains and plasmids used in this study. **Table S5.** Primers used in this study. **Figure S1.** SDS–PAGE of purified 2,3-DHBD. **Figure S2.** 2-HTA production by 2,3-DHBD_Ao mutants with single and double amino acid substitutions in the pure enzyme catalysis. ^a^Reaction conditions: 10 mM substrate, 1mg/mL protein, 3 M KHCO_3_, 5 mM MgCl_2_, 30 ℃, 200 rpm.

## Data Availability

The data sets used and/or analyzed during the current study are available from the corresponding author on reasonable request.
