# Supplementary material for: De novo biosynthesis of 2-hydroxyterephthalic acid, the monomer for high-performance hydroxyl modified PBO fiber, by enzymatic Kolbe–Schmitt reaction with CO2 fixation
Source: Biotechnol Biofuels Bioprod. 2023 Nov 20;16:179. doi: 10.1186/s13068-023-02413-0 (PMC10662693; doi:10.1186/s13068-023-02413-0)
Supplement: Supplementary file 1 — Additional file 1: Table S1. Enzymic carboxylation with natural substrates. Table S2. Quantitative analysis of protein expression in 2,3-DHBD—Ao, Aoopt, Fo, Foopt, Ao F27/T62. Table S3. Codon-optimized DNA sequences. Table S4. Strains and plasmids used in this study. Table S5. Primers used in this study. Figure S1. SDS–PAGE of purified 2,3-DHBD. Figure S2. 2-HTA production by 2,3-DHBD_Ao mutants with single and double amino acid substitutions in the pure enzyme catalysis. aReaction conditions: 10 mM substrate, 1mg/mL protein, 3 M KHCO3, 5 mM MgCl2, 30 ℃, 200 rpm. [file 13068_2023_2413_MOESM1_ESM.docx]

**Additional Information**

**De novo biosynthesis of 2-hydroxyterephthalic acid, the monomer for high-performance hydroxyl modified PBO fiber, by enzymatic Kolbe-Schmitt reaction with CO_2_ fixation**

Yali Zhou^a^, Shiding Zhang^a^, Shiming Huang^a^, Xuanhe Fan^a^, Haijia Su^a^, Tianwei Tan^a*^

^a^National Energy R&D Center for Biorefnery, Beijing Key Lab of Bioprocess, College of Life Science and Technology, Beijing University of Chemical Technology, No. 15 North 3rd Ring Rd East, 100029 Beijing, PR China.

*Correspondence:twtan@mail.buct.edu.cn

**Purification of recombinant proteins**

2,3-DHBD genes were cloned into the vector pET-28a(+) with His_6_-tag. *E. coli* BL21(DE3) strains expressed the protein and were ultrasonically broken to get crude enzyme solution. Ni^2+^ affinity chromatography was used for purification. Protein was analyzed by SDS polyacrylamide gel electrophoresis (SDS-PAGE) with 15% polyacrylamide gel. The protein content was measured by protein UV quantification the NanoPhotometer-N60.

**Table S1** Enzymic carboxylation with natural substrates ^a^

| Entry | Enzyme ^b^ | Resorcinol conversion (%) | | 2,6-dihydrobenzoic acid selectivity (%) | 2,4-dihydrobenzoic acid selectivity (%) |
| --- | --- | --- | --- | --- | --- |
| 1 | Control | | 0 | 0 | 0 |
| 2 | 2,3-DHBD_Ao | | 46.05 | 55.13 | 11.36 |
| 3 | 2,3-DHBD_Ao_opt_ | | 52.80 | 53.27 | 8.57 |
| 4 | 2,3-DHBD-Fo | | 50.06 | 38.17 | 22.51 |
| 5 | 2,3-DHBD-Fo_opt_ | | 39.87 | 21.05 | 13.72 |
| 6 | SAD-Tm | | 59.98 | 11.73 | 2.04 |
| 7 | SAD-Tm_opt_ | | 53.87 | 28.25 | 3.64 |

^a^ Reaction conditions: 30 mg/mL lyophilized whole cells, 10 mM substrate, 3 M KHCO_3_, 30℃, 180 rpm, 24 h.

^b^ Enzyme: 2,3-DHBD_Ao, 2,3-Dihydroxybenzoate decarboxylase from *Aspergillus oryzae* ; 2,3-DHBD_Fo, 2,3-Dihydroxybenzoate decarboxylase from *Fusarium oxysporum* ; SAD-Tm, salicylic acid decarboxylase from *Trichosporon moniliiforme*; opt, codon-optimized.

**Table S2** The quantitative analysis of protein expression in 2,3-DHBD- Ao, Ao_opt_, Fo, Fo_opt_, Ao ^F27/T62^

| Strains | 2,3-DHBD-Ao | 2,3-DHBD-Ao_opt_ | 2,3-DHBD-Fo | 2,3-DHBD-Fo_opt_ | 2,3-  DHBD-  Ao^F27G/T62A^ |
| --- | --- | --- | --- | --- | --- |
| Protein  (mg/mg cell) | 0.064±0.0072 | 0.035±0.0054 | 0.056±0.0036 | 0.071±0.0088 | 0.073±0.0081 |


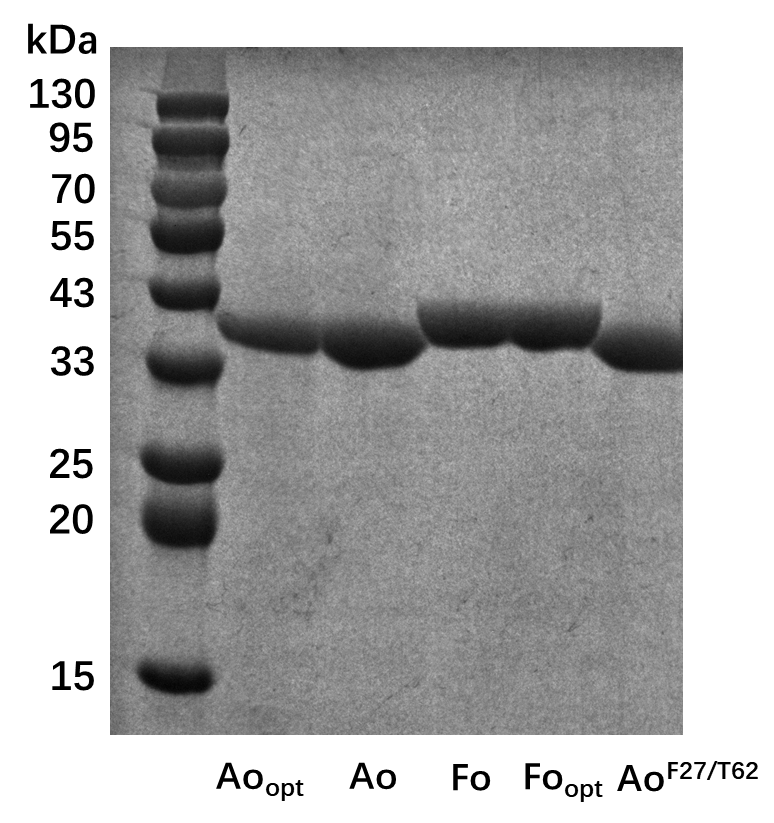


**Figure S1** SDS-PAGE of purified 2,3-DHBD

**Table S3** The codon-optimized DNA sequences

| Enzyme | DNA sequences |
| --- | --- |
| 2,3-DHBD_Ao_opt_ | |
|  | ggatccatgctgggtaaaattgccctggaagaagcctttgcactgccgcgctttgaagaaaaaacccgttggtgggcaagtctgtttagtaccgatgccgaaacccatgttaaagaaattaccgatattaacaagatccgtattgaacatgcagataaacatggcgttggctatcagattctgagttataccgcaccgggcgtgcaggatatttgggaccctgtggaagcccaggccctggcagttgaaattaatgattatattgccgaacaggtgcgcgtgaatccggatcgctttggtgcatttgccaccctgagcatgcataatccgaaagaagcagccgatgaactgcgtcgctgcgttgaaaaatatggctttaaaggtgccctggtgaatgatacccagcgtgccggtccggatggtgacgatatgattttctatgataatgcagattgggatatcttttggcagacctgtaccgaactggatgttccgttttatatgcatccgcgcaatccgaccggtaccatctatgaaaaactgtgggcagatcgcaaatggctggttggcccgccgctgagttttgcccatggtgtgagcctgcatgttctgggtatggtgaccaatggcgtttttgatcgccatccgaaactgcagattattatgggccatctgggtgaacatgtgccgtttgatatgtggcgtattaatcattggtttgaagatcgcaaaaagctgctgggtctggcagaaacctgcaaaaagactattcgtgattattttgcagagaatatctggattaccaccagcggtcattttagtaccaccaccctgaatttttgcatggcagaagttggcagtgatcgcattctgtttagcattgattatccgtttgaaacctttagcgatgcctgcgaatggtttgataatgccgaactgaatggtaccgatcgcctgaaaattggccgtgaaaatgccaaaaaactgtttaaactggatagttataaggacagcagcgcctaagtcgac |
| 2,3-DHBD_Fo_opt_ | |
|  | gtatgctgggcaaagttgcactggaagaagcctttgccctgccgcgtcataaagaacgcacccgttggtgggccggcctgggtgccattgatccggataaacatgccgcagaaattaatgatattaccgaacagcgtattaagtatatgaatgaacatggcgttggctataccattctgagttataccgcaccgggtgtgcaggatgtttgggatccgaaagaagcacaggcactggccgttgaagttaatgattatattgccgatgcaattaaggcacatccggatcgtctgggtgcctttgcaaccctgagtatgcatgatccgaaagaggccgccgaagaactgcgtcgcgttgtgaccaaatatggttttaaaggtgccctggttaatgatacccagcgtgcaggcgcagatggcgatgatatgattttctatgatggcccggaatgggatgttttctggagcaccgtgaccgatctggatgttccgttttatctgcatccgcgcaatccgaccggcagtattcatgaaaaactgtgggccaaacgcagttggctgattggcccgccgctgagttttgcccagggcgtgagtctgcatgcactgggcatggtgaccaatggcgtgtttgatcgccatccgaaactgcagattgttctgggtcatctgggtgaacatattccgtttgatatgtggcgtattaatcattggtttgaagatattaagaagccgctgggtctgagctgcaaactgaccattcgcgaatattttgcccgtaatctgtggattaccaccagcggtcattttagtaccagtaccctgcagttttgcctgggtgaagttggtgccgatcgtattctgtttagcattgattatccgtttgaaaatttcagcgatgcatgtacctggtatgatggtctggccattaatgatgttgataaacgcaaaattggcaaagataatgccaaaaaactgtttaaactgccgcagttttatcagagcgaagat |
| SAD_Tm_opt_ | |
|  | atgcgcggcaaggtttctctcgaggaggctttcgagctccccaagtacgcggcgcagaccaagcagaaggccgagctgtacatcgcgcccaacaaccgcgaccggtactttgaggagattctgaacccgtgcggcaaccgcctcgagctctccaacaagcacggaatcggctacaccatctactcgatctactctcctggcccgcaggggtggacggaccggaaggagtgcgaggcgtacgctcgcgagtgcaacgacttcatccacagcgagatctccaagcacaaggaccgcatgggcgcgttcgccgcgctctcgatgcacgaccccaagcaggctagcgaggagctcacccgctgcgtcaaggagctcggcttcctcggtgcgctcgtaaacgacgtccagcacgctggtcccgagggcgaggagtatatcttctacgaccagcccgagtgggacattttctggcagacttgcgtcgacctcgacgtcccattctacctccaccccgagccacccgtcggttcgtactttaagcagcagtacgagggccgcaagtacctcatcggtccccccgtgagcttcgccaacggtgtctcgctccacctcctcggcatgatcgtcaacggtgtctttgaccgcttccccaagctcaaggtcatcctcggccacctcggcgagcacatcccgggtgacttctggcgcatcgagcactggttccagcactgctcgcgccccctcgccgagtcccgcggcgatgtcttcgccaagcaccccctcctgcactacttccgcaacaacatctggctcaccacctcgggcaacttctctacggagacgctcaagttctgcgtggaccacgtcggtgccgaccgtgtcctcttctccgtcgactcgccctatgagcacattgacgtcggctgcgggtggtacgacgacaacgccaagcagatcatggaggccgtcggcggcgagaaggcctacaaggacattggccgtgacaacgccaagcgtctgttcaagctcggcaacttctacgactcggaggcgtag |

In the pure enzyme catalysts system, 10 mM substrate, 1mg/mL purified enzyme protein, 3 M KHCO_3_ and 5mM MgCl_2_ were added into 1 mL deionized water. The mixture was incubated at 30℃ for 48 h in sealed glass vials. Single mutants of F27G, T62A and double mutant F27G/T62A were 6.8-fold, 8.0-fold and 22.8-fold higher than the wild-type.

**Figure S2** 2-HTA production by 2,3-DHBD_Ao mutants with single and double amino acid substitutions in the pure enzyme catalysis.

^a^Reaction conditions: 10 mM substrate, 1mg/mL protein, 3 M KHCO_3_, 5mM MgCl_2_, 30℃, 200 rpm.

**Table S4** Strains and plasmids used in this study

| **Strains and plasmids** | **Description** | **Source** |
| --- | --- | --- |
| **Plasmids** | | |
| pET-28a(+) | ColE1 ori, Kan^R^, *lacI*, P_T7_ | Lab stock |
| pET-2,3-DHBD_Ao | pET-28a(+), P_T7_ *2,3-DHBD_Ao* | This study |
| pET-2,3-DHBD_Ao_opt_ | pET-28a(+), P_T7_ *2,3-DHBD_Ao_opt_* | This study |
| pET-2,3-DHBD-Fo | pET-28a(+), P_T7_ *2,3-DHBD_Fo* | This study |
| pET-2,3-DHBD-Fo_opt_ | pET-28a(+), P_T7_ *2,3-DHBD_Fo_opt_* | This study |
| pET-SAD-Tm | pET-28a(+), *SAD-Tm* | This study |
| pET-SAD-Tm_opt_ | pET-28a(+), *SAD-Tm_opt_* | This study |
| pET-2,3-DHBD_Ao^F27G^ | pET-28a(+), P_T7_ *2,3-DHBD_Ao^F27G^* | This study |
| pET-2,3-DHBD_Ao^T62A^ | pET-28a(+), P_T7_ *2,3-DHBD_Ao^T62A^* | This study |
| pET-2,3-DHBD_Ao^F27G/T62A^ | pET-28a(+), P_T7_ *2,3-DHBD_Ao^F27G/T62A^* | This study |
| pSP-GM1 | 2μm ori, Amp^R^, *URA3*, P_TEF1_-T_ADH1_, P_PGK1_-T_CYC1_ | Partow et al. (2010)[1] |
| pSP-Hyg5 | pSP-GM1, P_PGK1_-*Hyg5*-T_CYC1_ | This study |
| pSP-2,3-DHBD_Ao | pSP-GM1, P_TEF1_-*2,3-DHBD_Ao*-T_ADH1_ | This study |
| pSP-Hyg5-2,3-DHBD_Ao | pSP-GM1, P_PGK1_-*Hyg5*-T_CYC1_, P_TEF1_-*2,3-DHBD_Ao*-T_ADH1_ | This study |
| pSP-Hyg5-2,3-DHBD_Ao^T62A^ | pSP-GM1, P_PGK1_-*Hyg5*-T_CYC1_, P_TEF1_-*2,3-DHBD_Ao^T62A^*-T_ADH1_ | This study |
| pSP-Hyg5-2,3-DHBD_Ao^F27G/T62A^ | pSP-GM1, P_PGK1_-*Hyg5*-T_CYC1_, P_TEF1_-*2,3-DHBD_Ao* *^F27G/T62A^*-T_ADH1_ | This study |
| **Strains** | | |
| *E. coli* Trans 10 | F^-^ *mcr*A Δ(*mrr-hsd*RMS-*mcr*BC) φ80 *lac*ZΔM15Δ *lac*X74 *rec*A1 *ara*Δ139Δ(*ara*-*leu*)7697 *gal*U *gal*K *rps*L (Str^R^)*end*A1 *nup*G | TransGen Biotech |
| *E. coli* BL21(DE3) | F^-^ *omp*T *hsd*S_B_ (r_B_^-^ m_B_^-^) *dcm*(DE3) | TransGen Biotech |
| CEN.PK 113-5D | *MATa SUC2 MAL2-8c ura3-52* | Chen Y. et al.(2012)[2] |
| S288C-AT | *MATa SUC2 gal2 mal2 mel flo1 flo8-1 hap1 ho bio1 bio6 Δura3 Δaro7 Δtrp3* | Lab stock |
| BL-0 | *E. coli* BL21(DE3), pET-28a(+) | This study |
| BL-A | *E. coli* BL21(DE3), pET-*2,3-DHBD_Ao* | This study |
| BY-01 | BY4741, pSP-GM1 | This study |
| BY-A | BY4741, pSP-2,3-DHBD_Ao | This study |
| 5D-H | CEN.PK 113-5D, pSP-*Hyg5* | This study |
| 5D-HA | CEN.PK 113-5D, pSP-*Hyg5*-*2,3-DHBD_Ao* | This study |
| SC-01 | S288C-AT, pSP-GM1 | This study |
| SC-H | S288C-AT, pSP-*Hyg5* | This study |
| SC-HA | S288C-AT, pSP-*Hyg5*-*2,3-DHBD_Ao* | This study |
| SC-HA1 | S288C-AT, pSP-*Hyg5*-*2,3-DHBD_Ao^T62A^* | This study |
| SC-HA2 | S288C-AT, pSP-*Hyg5*-*2,3-DHBD_Ao^F27G/T62A^* | This study |

**Table S5** Primers used in this study

| **Primer** | **Sequence (5' to 3')** | | **Description** | |  |
| --- | --- | --- | --- | --- | --- |
| **Plasmid construction** | | |  | |  |
| pET-Hyg5 construction | | |  | |  |
| pET-BamHI-Hyg5-F | | CGCGGATCCAGGAGATATACCATGAACCCGTCATCGCTTG | | BamHI | |
| pET-HindIII-Hyg-R | | CCCAAGCTTCTACATGACCACGCCCTCG | | HindIII | |
| pSP-GM1-Hyg5 construction | | | |  | |
| pSP-BamHI-hyg5-F | | CGGGATCCATGAACCCGTCATCGCTTGT | | BamHI | |
| pSP-HindIII-hyg5-R | | CCCAAGCTTCTACATGACCACGCCCTCGAT | | HindIII | |
| pGM1-2,3-DHBD_Ao/pGM1-Hyg5-2,3-DHBD_Ao construction | | | |  | |
| pSP-SpeI-A-F | | GGACTAGTATGCTCGGTAAGATCGCTCTCG | | SpeI | |
| pSP-PacI-A-R | | CCTTAATTAACTAAGCTGAACTATCCTTGTAAGAATCAAGCT | | PacI | |
| 2,3-DHBD_Ao site-directed mutagenesis | | | | Gibson |  |
| F27G Gib-F | | GCAAGTCTCGGTTCCACGGACGCCGAAACCCACGTCA | |  | |
| F27G Gib-R | | CGTGGAACCGAGACTTGCCCACCAGCGG | |  | |
| T62A Gib-F | | TCATACGCTGCACCCGGTGTACAAGACA | |  | |
| T62A Gib-R | | CGGGTGCAGCGTATGAGAGGATTTGGTAGCCGA | |  | |
| **Plasmid verification** | | | |  | |
| pET-T7 | |  | |  | |
| pET-T7-F | | ACTGGTTTCACATTCACCACCC | |  | |
| pET-T7-R | | AACAAGAGTCCACTATTAAAGAACGTGG | |  | |
| pSP-GM1 | | |  | |  |
| P TEF-F | | CCTCTTTCTTCCTCTAGGGTGTCG | |  | |
| T ADH1-R | | ACCTGAGAAAGCAACCTGACCTAC | |  | |
| T CYC-R | | GTGGATAACCGTATTACCGCCTT | |  | |

The primers used in this study were synthesized by BGI (China)

**References**

1. Partow S, Siewers V, Bjørn S, Nielsen J, Maury J. Characterization of different promoters for designing a new expression vector in Saccharomyces cerevisiae. Yeast. 2010;27(11):955-64.

2. Chen Y, Partow S, Scalcinati G, Siewers V, Nielsen J. Enhancing the copy number of episomal plasmids in Saccharomyces cerevisiae for improved protein production. FEMS Yeast Research. 2012;12(5):598-607.
